# Changes in the diagnostic trajectory of transthyretin cardiac amyloidosis over six years

**DOI:** 10.1007/s00380-024-02408-3

**Published:** 2024-05-06

**Authors:** Anouk Achten, Vanessa P. M. van Empel, Jerremy Weerts, Sanne Mourmans, Fabienne Beckers-Wesche, Mireille Spanjers, Arno Gingele, Hans-Peter Brunner-La Rocca, Sandra Sanders-van Wijk, Christian Knackstedt

**Affiliations:** 1 Department of Cardiology, Cardiovascular Research Institute Maastricht (CARIM), Maastricht University Medical Centre (MUMC+), PO Box 5800, 6202 AZ Maastricht, The Netherlands; 2 Department of Cardiology, Zuyderland Medical Centre, Heerlen, The Netherlands

**Keywords:** Cardiac amyloidosis, Diagnostic trajectory, Diagnostic delay, Heart failure, Transthyretin amyloidosis

## Abstract

Awareness of transthyretin amyloid cardiomyopathy (ATTR-CM) has increased over the years due to diagnostic and therapeutic developments. Timely initiation of novel disease-modifying treatments improves both morbidity and mortality, which underlines the necessity for a prompt diagnosis. Nevertheless, early diagnosis of ATTR-CM remains challenging. This is a retrospective observational cohort study of patients diagnosed with ATTR-CM. Between 2016 and 2023, 87 patients were diagnosed with cardiac amyloidosis of which 65 (75%) patients with ATTR-CM and 22 (25%) patients with light chain amyloidosis. This study included 65 ATTR-CM patients (mean age 77 ± 7 years; 86% male) of whom 59 (91%) with wild-type ATTR-CM (ATTRwt) and six (9%) with variant ATTR-CM. We observed a surge in ATTR-CM diagnoses from 3 patients/year (2016–2020) to 16 patients/year (2021–2023), driven by ATTRwt. Nevertheless, the interval between the onset of heart failure symptoms and ATTR-CM diagnosis has not changed significantly (2016–2020 27.3 months [18.6–62.4]; 2021–2023 30.0 months [8.6–57.2]; *p* = 0.546), driven by time to referral. Red flags for ATTR-CM preceded diagnosis by several years: left ventricular hypertrophy (79%, 5.8 years [3.3–7.0]) and carpal tunnel syndrome (49%, 6.8 years [2.3–12.1]). Despite the presence of typical red flags, symptom-to-diagnosis duration has remained similar driven by time to referral. Improved recognition of red flags for ATTR-CM could reduce the time to diagnosis and improve overall recognition.

**Supplementary Information:**

The online version contains supplementary material available at 10.1007/s00380-024-02408-3.

## Introduction

Amyloidosis is a spectrum of diseases in which misfolded proteins aggregate into amyloid fibrils, causing extracellular depositions in various tissues and organs. Cardiac amyloidosis (CA) is characterized by depositions of these insoluble amyloid fibrils in the interstitial space between cardiac myocytes, leading to left ventricular hypertrophy (LVH), heart failure and conduction disorders. Amyloid formation can be caused by various proteins, but two precursor proteins alone cause up to 98% of CA cases [1]. One of these proteins is transthyretin (TTR), a serum transporter protein synthesised by the liver. Rarely, TTR amyloid fibrils are formed due to a mutation in the TTR gene (variant); more commonly, it is due to unresolved reasons related to ageing (wild-type). Second, in cardiac light chain amyloidosis (AL-CA), amyloid fibrils are created from abnormal light chains generated by monoclonal plasma cells [1].

Transthyretin amyloid cardiomyopathy (ATTR-CM) was long thought to be a rare disease. However, awareness of ATTR-CM has improved as a result of non-invasive diagnostic and disease-modifying therapeutic developments, and recent studies have suggested that it is more common than previously anticipated. The prevalence of ATTR-CM has been reported to vary between 6 and 25% in diverse population cohorts [2–7]. At this moment, ATTR-CM progression can only be mitigated rather than completely stopped or even reversed. Consequently, timely initiation of novel disease-modifying treatments is required at early stages of ATTR-CM to improve morbidity and mortality, which strikes the necessity for a prompt and early diagnosis [8–10]. Nevertheless, early diagnosis of ATTR-CM remains challenging and diagnosis is often delayed [11–13]. Delayed diagnosis of ATTR-CM can be attributed to several factors, including unawareness of the disease, heterogenic and non-specific symptoms at early stages, and overlapping conditions with subsequent misdiagnosis [14, 15].

ATTR-CM may occur as an isolated disease, but more commonly it is part of a multisystemic disorder with several extra-cardiac manifestations [1]. Recognition of early cardiac and extra-cardiac manifestations may provide a more timely diagnosis and earlier therapy initiation. Nevertheless, it is uncertain as to whether increased awareness of cardiac and extra-cardiac manifestations of ATTR-CM has contributed to a reduction in the diagnostic delay of ATTR-CM over the years or if it has solely resulted in an augmentation of ATTR-CM diagnoses. Multiple studies have presented conflicting findings on this matter [3, 11, 16, 17]. Moreover, it is known that the translation of scientific advancements into practical application takes time, especially within smaller healthcare institutions [18]. Notably, the European Society of Cardiology (ESC) published its position paper in 2021 [1], potentially leading further to heightened awareness and possible reductions in diagnostic delay of ATTR-CM after 2021.

Acknowledging the importance of recognizing and overcoming diagnostic challenges for cardiac amyloidosis, this study evaluates the frequency and diagnostic trajectory of ATTR-CM in a tertiary medical centre in the Netherlands, with emphasis on improving time to diagnosis. Moreover, analyses were conducted to examine the clinical characteristics of patients and the presence of typical red flags associated with ATTR-CM, with a comparative assessment between patients diagnosed before and after publication of the ESC position paper and between the wild-type (ATTRwt) and variant (ATTRv) forms of ATTR-CM.

## Methods

### Study population, consent, and technical examination

In this single-centre retrospective observational study, all patients diagnosed with ATTR-CM from 2016 until 2023 were included. Patients with ATTRv but no cardiac involvement were excluded. The study was approved by the medical ethics committee of the Maastricht University Medical Centre (non-WMO 2022-3440) and adhered to the principles outlined in the Declaration of Helsinki. Diagnosis of ATTR-CM was based on positive bone scintigraphy (Perugini grade II or III) in combination with absence of monoclonal gammopathy, and/or positive endomyocardial biopsy (histology and immunohistochemistry) and/or CA characteristics on cardiac MRI in combination with known ATTR genotype, conform recommendations [1, 19]. Bone scintigraphy was performed using Tc-99m hydroxymethylene diphosphonate tracer and myocardial tracer uptake was visually graded according to semi-quantitative Perugini stages as grade 0, 1, 2 or 3 [20]. All patients diagnosed with ATTR-CM underwent subsequent genetic testing. Patients’ characteristics and clinical data were retrieved from electronic health records. All data processed were aligned with the Maastricht Cardiomyopathy registry infrastructure [21].

At baseline, all patients underwent laboratory testing, transthoracic echocardiography and electrocardiography as part of the routine clinical assessment. The baseline was delineated as the point of diagnosis for ATTR-CM. Multiple electrocardiographic abnormalities were assessed by one clinical researcher (A.A.). Micro voltage was considered present when the amplitudes of all the QRS complexes in the limb leads were less than five mm, or when the amplitudes of all the QRS complexes in the precordial leads were less than ten mm. LVH on ECG was assessed according to Sokolov-Lyon and Cornell criteria. Lastly, pseudo infarct pattern was defined as a pathological Q wave without previous myocardial infarction. A commercially available transthoracic echocardiography system was used to conduct the echocardiography. Cardiac chamber quantification, cardiac function and diastolic dysfunction were clinically evaluated as recommended [22]. Presence of typical cardiac and extracardiac manifestations, red flags of ATTR-CM were scored. Red flags included increased echocardiographic left ventricular wall thickness ≥ 12 mm, aortic valve stenosis, carpal tunnel syndrome, polyneuropathy, spinal canal stenosis, orthostatic complaints, presence of a pacemaker, conduction delay (PQ duration ≥ 200 ms or QRS duration ≥ 120 ms) or micro voltages on electrocardiography [1].

All patients underwent regular 6–12 months follow-up at our centre. The severity of CA at diagnosis was subdivided by the Gilmore staging criteria [23].

### Diagnostic pathway

Time between presentation with heart failure symptoms at the cardiology outpatient clinic and initiation of CA diagnostics, as well as the subsequent ATTR-CM diagnosis was obtained from the electronic health records. The time preceding the diagnostic process, referred to as the pre-diagnostic delay, was defined as the time in months between onset of heart failure symptoms and initiation of CA diagnostics. The initiation of CA diagnostics was determined as the initial date when a bone scintigraphy, cardiac MRI, or monoclonal M-protein serum test was conducted, with a focus on diagnosing CA. Subsequently, the diagnostic trajectory was delineated as the period between the initiation of CA diagnostics and the diagnosis of ATTR-CM.

To assess the diagnostic pathway over time, a comparison was made between two time periods, 2016–2020 and 2021–2023. These time periods were determined based on the publication of the ESC consensus paper in 2021, with the initial time interval characterizing the era preceding heightened awareness, therapeutic alternatives, and non-invasive diagnostic modalities. Furthermore, time between ATTR-CM diagnosis and various cardiac and extra-cardiac manifestations prior to CA diagnosis was extracted from the medical records.

### Statistics

Descriptive analyses were performed to compare baseline demographics, comorbidities, clinical and biological data, and echocardiographic measurements between groups based on diagnosis time period and ATTR-CM type. Furthermore, duration of different phases of the diagnostic trajectory were compared between the two time periods.

Categorical variables, presented as count and percentage, were compared using the Fisher exact test. Continuous variables were expressed as mean ± standard deviation (SD) if normally distributed (according to visual assessment of QQ-plots and Levene’s test of equal variances) and differences were tested using the students T test. Continuous variables were expressed as median and quartiles if not normally distributed, and tested using the Mann–Whitney *U* test. Finally, ordinal variables were represented as medians and [quartiles] and subjected to Mann–Whitney *U* test. All statistical analyses were performed using the statistical program IBM SPSS statistics software version 28 (SPSS Inc, Chicago, IL). A *p* value of less than 0.05 was considered statistically significant.

## Results

### Clinical characteristics

**Fig. 1:**
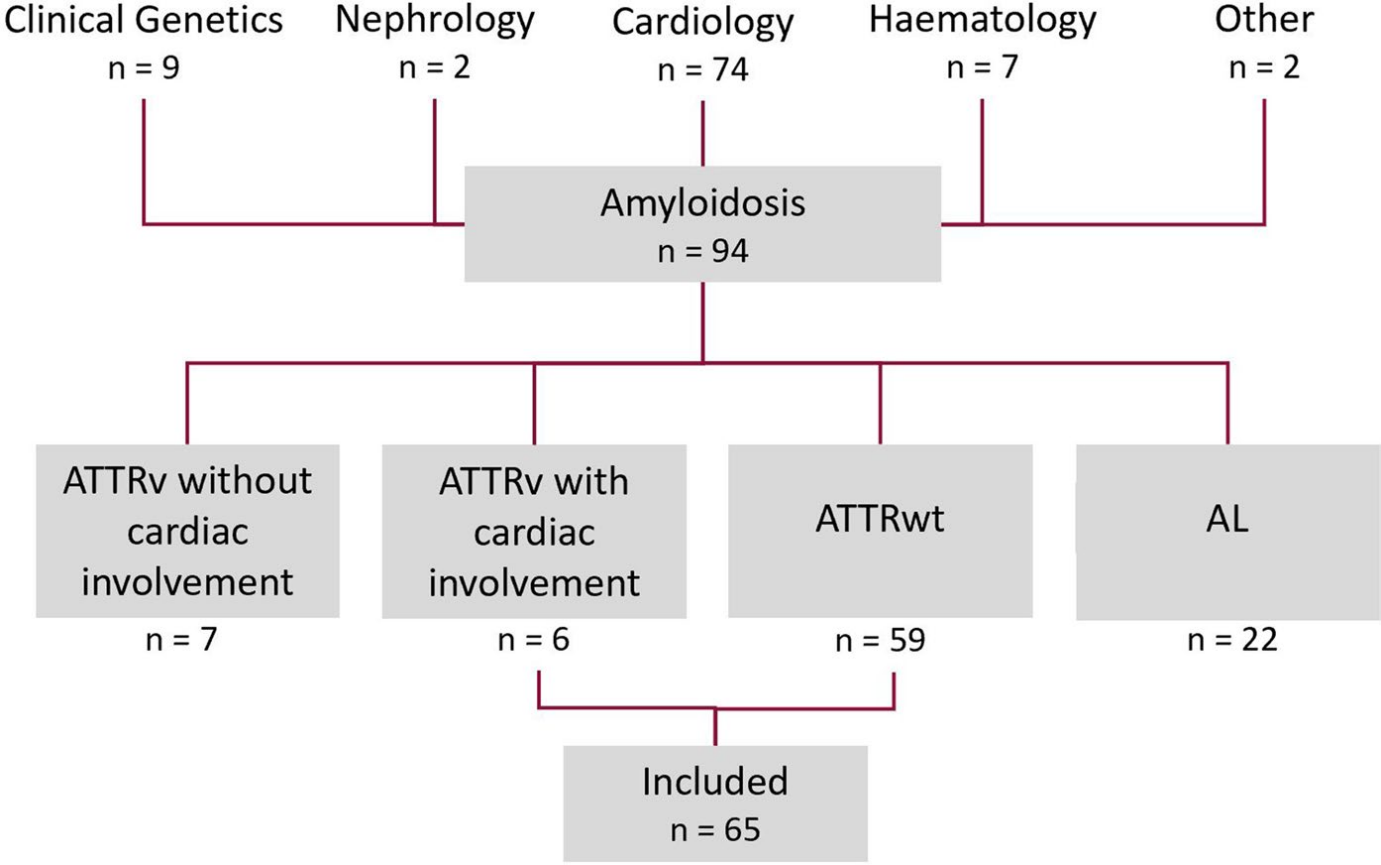
Flow diagram of amyloidosis patients. Abbreviations: ATTRwt, wild-type transthyretin amyloidosis; ATTRv, variant transthyretin amyloidosis, AL, amyloidosis light chain

Between 2016 and 2023, a total of 94 patients were diagnosed with amyloidosis (Fig. 1), of which 65 (69%) patients were diagnosed with ATTR-CM (mean age 77 ± 7 years; 86% male) and 22 (23%) patients with AL-CA (mean age 72 ± 9 years; 73% male). The remaining seven (8%) patients were ATTRv carriers, but did not manifest cardiac involvement on cardiac MRI, bone scintigraphy or cardiac biopsy. The majority of all amyloidosis patients were referred from the cardiology outpatient clinic (*n* = 74; 79%). Nine patients were referred subsequent to a confirmed ATTR genotype by the clinical genetics department (Fig. 1). Only patients diagnosed with ATTR and cardiac involvement (*n* = 65) were included for the subsequent analyses.

Out of the 65 ATTR-CM patients, 59 (91%) were diagnosed with ATTRwt (median age 78 years; 92% male), versus six (9%) with ATTRv (median age 71 years; 33% male). Monoclonality was observed in 12 (19%) patients with a median kappa to lambda ratio of 1.2 [0.90–1.60] and biopsies were performed in seven patients (six cardiac, one bone marrow and one fat pad biopsy), all of which were negative for light chain amyloidosis. Eventually, all 12 patients were diagnosed with monoclonal gammopathy of unknown significance by the haematologist, excluding AL-CA.

Patients diagnosed with ATTRwt were significantly older and more likely male compared to patients diagnosed with ATTRv (Supplemental Table S1). Overall, a significant proportion (40%) of ATTR-CM patients initially presented with advanced heart failure based on New York Heart Association classification class ≥ III (Table 1). Furthermore, at diagnosis 36% (*n* = 23) of ATTR-CM patients presented in prognostic stage I of the Gillmore staging system [23], while 50% (*n* = 33) presented in the more advanced prognostic stages II and III (Table 2).

**Table 1:** Clinical characteristics of patients diagnosed with transthyretin cardiac amyloidosis

Characteristic	Overall *n* = 65	Valid, *n*	2016–2020 *n* = 17 (26%)	Valid, *n*	2021–2023 *n* = 48 (74%)	*p*-Value
Male sex	56 (86%)	17	13 (77%)	48	43 (90%)	0.225
Age (years)	77 (± 7)	17	73 (± 9)	48	79 (± 6)	0.024*
ATTRwt	59 (91%)	17	13 (77%)	48	46 (96%)	0.036*
Medical history						
Diabetes mellitus type II	11 (17%)	17	1 (6%)	48	10 (21%)	0.263
Kidney disease	27 (42%)	17	7 (41%)	46	20 (42%)	1.000
Atrial fibrillation/flutter	44 (68%)	17	8 (47%)	48	36 (75%)	0.068
Hypertension	35 (54%)	17	11 (65%)	48	24 (50%)	0.398
Significant CAD	20 (31%)	17	5 (29%)	46	15 (31%)	1.000
Stroke	6 (9%)	17	3 (18%)	48	3 (6%)	0.179
NYHA class		16		45		0.602
NYHA I	2 (3%)		1 (6%)		1 (2%)	
NYHA II	33 (51%)		8 (47%)		25 (52%)	
NYHA III	24 (37%)		6 (35%)		18 (38%)	
NYHA IV	2 (3%)		1 (6%)		1 (2%)	
Perugini grade		9		44		0.433
Perugini 0	1 (2%)		0 (0%)		1 (2%)	
Perugini 1	2 (3%)		1 (6%)		1 (2%)	
Perugini 2	28 (43%)		5 (29%)		23 (48%)	
Perugini 3	22 (34%)		3 (18%)		19 (40%)	
Electrocardiography						
Heart rate (beats/min)	72.0 [63–84]	17	72 [62–82]	48	72 [63–84]	0.880
Abnormal axis	30 (46%)	13	6 (35%)	41	24 (50%)	0.644
Micro voltage	26 (40%)	11	4 (24%)	38	22 (46%)	0.306
High voltage	0 (0%)	11	0 (0%)	38	0 (0%)	1.000
QRS duration (ms)	107 [92–140]	13	118 [94–136]	41	104 [90–142]	0.693
QRS ≥ 120 ms	20 (31%)	13	6 (35%)	41	14 (29%)	0.909
PQ duration (ms)	206 [182–242]	10	199 [176–242]	19	208 [192–242]	0.383
PQ ≥ 200 ms	17 (26%)	10	5 (29%)	19	12 (25%)	1.000
Pseudo infarct pattern	24 (37%)	11	6 (35%)	35	18 (38%)	1.000
Delayed R wave propagation	27 (42%)	11	5 (29%)	30	22 (46%)	0.195
Abnormal repolarization	44 (68%)	13	11 (65%)	40	33 (69%)	1.000
Echocardiography						
LVEF (%)	50 [41–56]	17	46 [38–54]	48	50 [43–56]	0.247
IVSd (mm)	14 [12–16]	16	14 [12–16]	47	14 [12–16]	0.703
PWd (mm)	13 [11–15]	16	13 [12–15]	46	13 [11–15]	0.710
LVEDd (mm)	44 [41–50]	16	44 [43–48]	35	45 [41–51]	0.935
LVMI (g/m^2^)	122 [94–149]	15	131 [87–151]	29	120 [94–146]	0.816
LAVI (ml/m^2^)	55 [46–61]	15	52 [42–57]	35	56 [49–63]	0.156
e’ septal (cm/s)	4.8 [3.8–5.9]	12	5.0 [4.7–5.6]	32	4.8 [3.8–5.9]	0.429
e’ lateral (cm/s)	6.4 [5.7–7.8]	16	6.0 [5.5–7.7]	35	6.6 [5.7–8.1]	0.580
E/e’ average	13.9 [10.4–17.9]	10	11.5 [9.1–15.2]	31	14.6 [10.8–18.5]	0.133
TR velocity (m/s)	2.5 [2.2–2.8]	13	2.3 [2.1–2.5]	33	2.5 [2.3–2.9]	0.061
Laboratory testing						
eGFR (ml/min/1.73m^2^)	60.1 [43.2–74.5]	15	61.7 [56.6–76.8]	44	56.7 [37.7–71.0]	0.109
hsTnT (ng/l)	49 [33–72]	9	42 [26–52]	35	50 [37–86]	0.244
NT-proBNP (pg/ml)	2461 [1285–4110]	14	2588 [880–3941]	43	2461 [1315–4148]	0.831

Data are presented as *n* (%), mean ± SD or median [interquartile range]; *p < 0.050

Abbreviations: *ATTRwt* wild-type transthyretin amyloidosis, *ATTRv* hereditary transthyretin amyloidosis, *CAD* coronary artery disease, *NYHA* New York Heart Association, *LVEF* left ventricular ejection fraction, *IVSd* intraventricular septum diameter, *PWd* posterior wall diameter, *LVEDd* left ventricular end diastolic diameter, *LVMI* left ventricular mass index, *LAVI* left atrial volume index, *TR* tricuspid regurgitation, *eGFR* estimated glomerular filtration rate, *hsTNT* high sensitive troponin T, *NT-proBNP* N-terminal pro B-type natriuretic peptide

**Table 2:** Prognostic stage of patients at ATTR-CM diagnosis

Gillmore prognostic stage	Overall	2016–2020 *n* = 17	2021–2023 *n* = 48	*p*-Value
Stage I	23 (35%)	6 (35%)	17 (35%)	0.976
Stage IIa	8 (12%)	0 (0%)	8 (17%)	
Stage IIb	17 (26%)	7 (41%)	10 (21%)	
Stage III	8 (12%)	0 (0%)	8 (17%)	

Data are presented as *n* (%)

Stage I, NT-proBNP ≤ 3000 pg/ml & eGFR ≥ 45 ml/min

Stage IIa, NT-proBNP ≤ 3000 pg/ml & eGFR < 45 ml/min

Stage IIb, NT-proBNP > 3000 pg/ml & eGFR ≥ 45 ml/min

Stage III, NT-proBNP > 3000 pg/ml & eGFR < 45 ml/min

Abbreviation: *NT-proBNP* N-terminal pro B-type natriuretic peptide

Notably, 13 (20%) patients presented without LVH on echocardiography (left ventricular wall thickness < 12 mm; *n* = 3, 23%, ATTRv; *n* = 10, 77% ATTRwt). Among this subgroup, a notably lower but not statistically significant, proportion of patients presented in the more advanced prognostic stages II and III compared to patients with LVH: 23% versus 59%, respectively (*p* = 0.225).

### Red flags

**Fig. 2:**
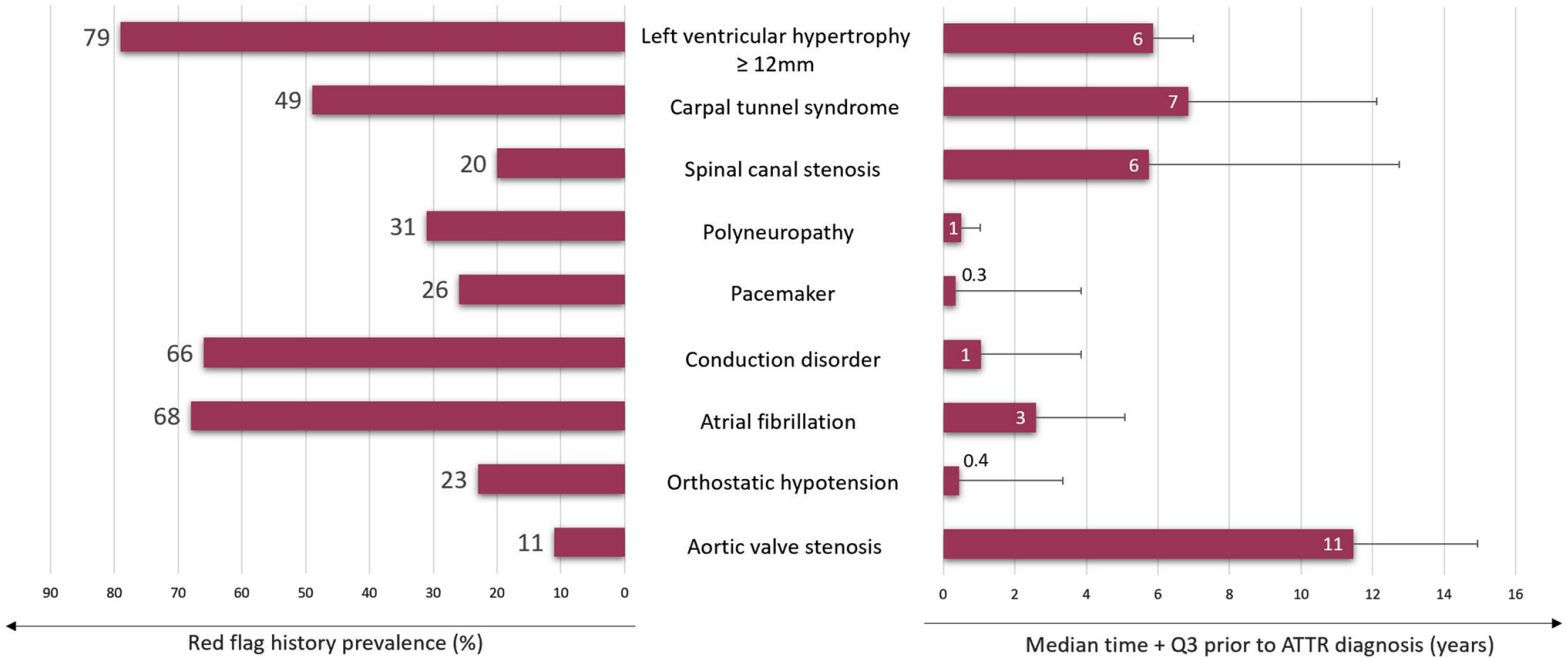
Prevalence and time lag of typical red flags prior to final ATTR diagnosis. Abbreviations: ATTR, transthyretin amyloidosis; Q3, third quartile

Typical cardiac and extra-cardiac manifestations of amyloid depositions were prevalent and presented years before the final ATTR diagnosis (Fig. 2). For instance, LVH (≥ 12 mm) and conduction disorders were depicted in 79% and 66% of the ATTR patients, preceding ATTR diagnosis with a median of 5.8 [3.3–7.0] and 1.1 [0.4–3.8] years, respectively. Furthermore, extra-cardiac findings, such as tenosynovial diseases (carpal tunnel syndrome and spinal stenosis) presented in 49% and 20% of patients, preceding diagnosis with a median of 6.8 [2.3–12.1] and 5.8 [1.5–12.7] years, respectively. An abnormal ECG was prevalent in 81% and preceded the final ATTR diagnosis with a median of 3.2 [0.6–8.2] years. None of the ATTR patients had LVH signs in their baseline ECG, whereas micro voltages were detected in the baseline ECG of 26 (40%) patients (Table 1). The median number of red flags for ATTR-CM was three.

### Diagnostic trajectory

**Fig. 3:**
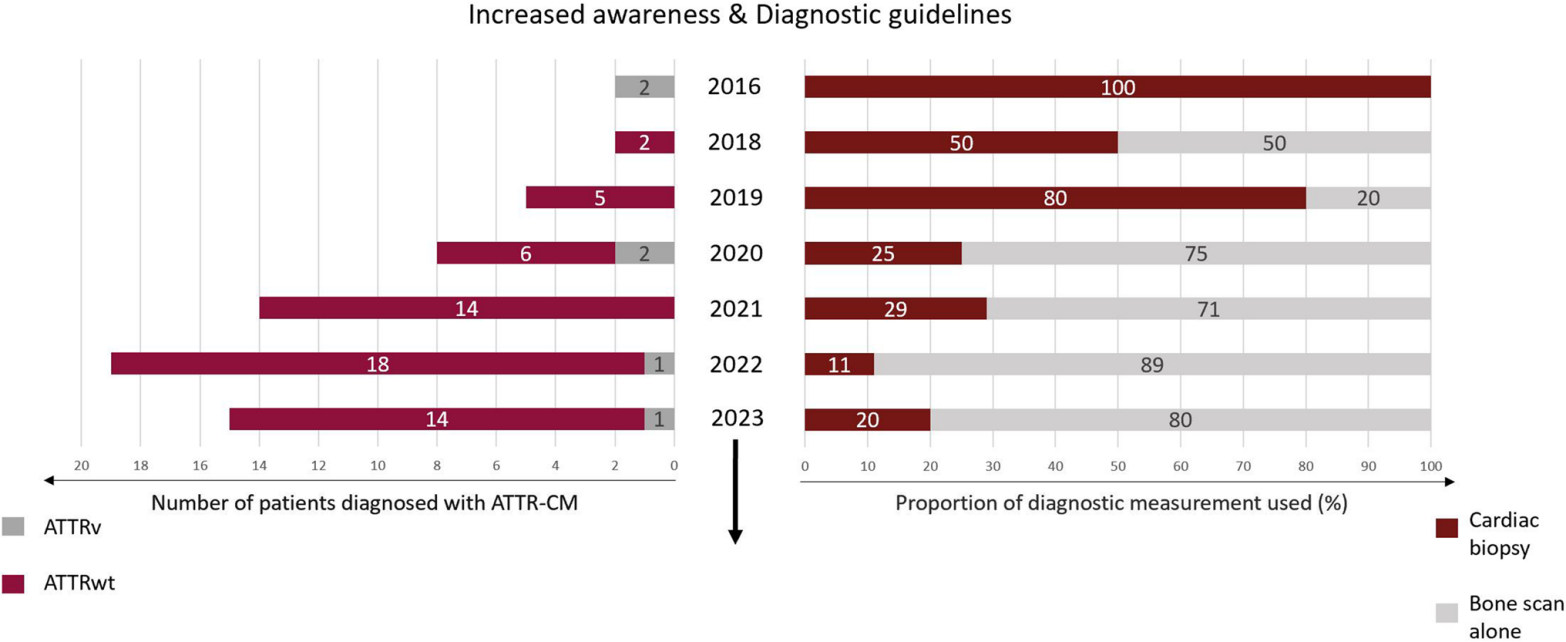
Number of yearly diagnosed ATTR patients and diagnostic methods employed. No patients were diagnosed in 2017; therefore, this year is not included in the figure. Abbreviations: ATTRwt, wild-type transthyretin amyloidosis; ATTRv, variant transthyretin amyloidosis

During the initial 5-year period from 2016 to 2020, 17 patients were diagnosed (*n* = 13 ATTRwt, *n* = 4 ATTRv), during the subsequent 3-year period (2021–2023) there was a notable increase in diagnoses with 48 patients being identified (*n* = 46 ATTRwt, *n* = 2 ATTRv) (Table 1). Additionally, the diagnostic approach shifted towards more non-invasive bone scintigraphy and fewer invasive cardiac biopsies. In the first 5-year period, cardiac biopsy was performed in 53% of the patients (9 out of 17). Usage of cardiac biopsy was reduced to 19% in the second 3-year period (*p* = 0.017). By the year 2022, bone scintigraphy alone was used to diagnose ATTR-CM in 91% of all cases (Perugini grade ≥ II, without monoclonality) (Fig. 3).

**Fig. 4:**
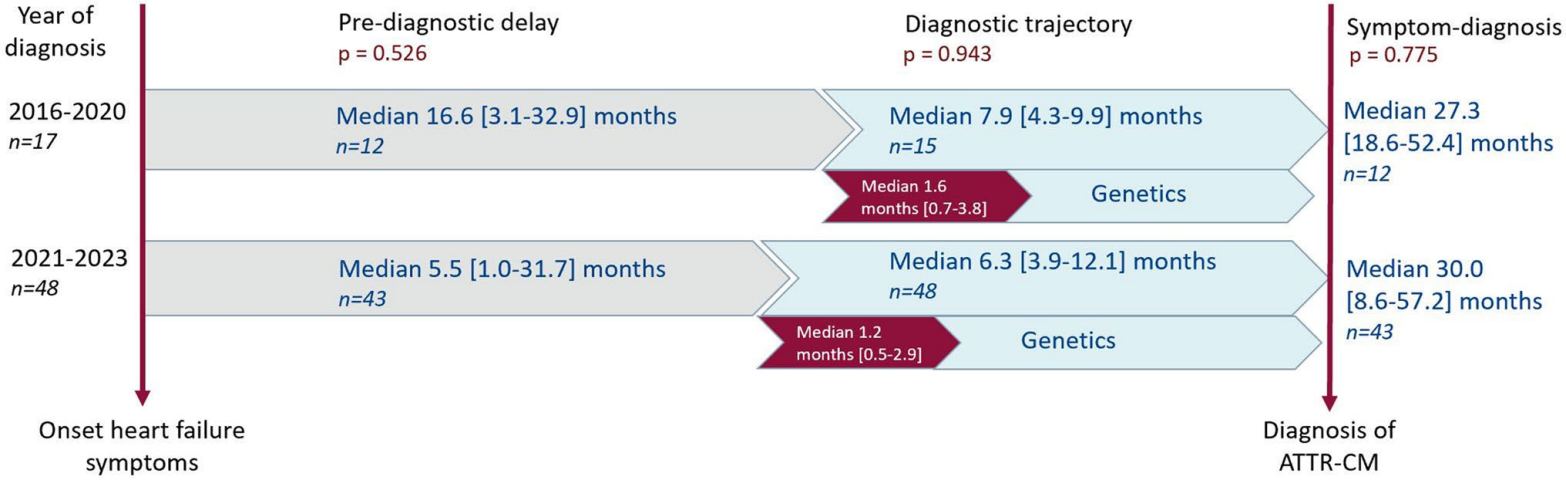
Duration of each phase in the diagnostic trajectory of cardiac amyloidosis. Abbreviation: ATTR-CM, cardiac transthyretin amyloidosis

Despite an increase in disease awareness and a less invasive diagnostic trajectory, we observed no statistically significant reduction in the median duration from the onset of heart failure symptoms to the final diagnosis (2016–2020 27.3 months [18.6–62.4]; 2021–2023 30.0 months [8.6–57.2]; *p* = 0.546). The pre-diagnostic delay formed the most prolonged segment of the overall symptom-to-diagnosis duration (median of 16.6 and 5.5 months pre-diagnostic delay vs median of 7.9 and 6.3 months diagnostic trajectory, respectively). The duration of the diagnostic trajectory itself was primarily determined by genetic testing (80% and 81% respectively of the diagnostic trajectory) (Fig. 4). When evaluating the duration of the diagnostic trajectory, specifically without the inclusion of genetic testing, we did not observe a significant shortening as well (1.6 months [0.7–3.8] vs 1.2 months [0.5–2.9]; *p* = 0.387; Fig. 4).

Although the duration from symptom onset to diagnosis was not statistically different per time period, it is noteworthy that 77% of patients who did not exhibit LVH on echocardiography, received their diagnosis in the latter portion of the recruitment period (2021–2023). Furthermore, there were no significant differences between ATTR-CM patients diagnosed in the first and the second time period, except for a slight increase in age and a higher occurrence of the wild-type form among patients diagnosed during the latter period (Table 1).

In assessing the diagnostic trajectory of patients presenting with three or more red flags indicative of ATTR-CM compared to those with fewer red flags, no statistically significant difference was observed in the duration of the diagnostic trajectory (*p* = 0.670).

## Discussion

We herein present the diagnostic trajectory, prevalence of typical red flags, and prognostic staging in patients diagnosed with ATTR-CM. This study highlights an increase in the number of diagnoses of ATTR-CM, primarily driven by ATTRwt. Additionally, the diagnostic approach shifted towards more non-invasive bone scintigraphy replacing the need for invasive cardiac biopsies in most cases. Diagnostic indicators such as LVH, conduction disorders, ECG abnormalities and tenosynovial disorders were highly prevalent and often preceded the ATTR-CM diagnosis by several years, indicating a prolonged and unnoticed progression of the disease. Despite an increase in number of ATTR-CM diagnoses and a less invasive diagnostic trajectory, the symptom-to-diagnosis duration has remained similar driven by time to referral. Prognostic staging revealed that a substantial proportion of ATTR-CM patients presented in advanced stages, indicative of worse survival rates.

ATTR-CM was historically recognised as a rare disease, and AL-CA was commonly regarded as the predominant etiological factor in CA. Awareness of ATTR-CM, however, has improved as a result of non-invasive diagnostic and disease-modifying therapeutic developments, and ATTR-CM has emerged as the predominant form of CA [11, 24]. Similar to observations made in numerous other medical centres [3, 11, 13, 16, 25], we have witnessed a remarkable surge in ATTRwt diagnoses over the past decade from 3 patients/year (2016–2020) to 16 patients/year (2021–2023). It is reasonable to anticipate further increase in the number of diagnoses of ATTR-CM, given the rise in general life expectancy (from 67 years in 2000 to 73 years in 2019) [26] and a prevalence of 25.0% of ATTR-CM in elderly populations [7]. Thus, suggesting inclusion of ATTR-CM in the differential diagnosis for routine clinical practice. But, this would also impose conducting a substantial number of bone scintigraphies, given the considerable size of the at-risk population. This underscores the necessity for enhancing screening strategies, in patients with suspicion of ATTR-CM and establishing a more precise pretest probability to mitigate unnecessary tests.

Although the number of diagnoses of ATTR-CM has increased over the years, diagnosis is frequently established in the relatively late disease stages, especially for ATTRwt [8]. The causes for this diagnostic delay are multifactorial and include symptom overlap with other conditions causing frequent misdiagnoses, heterogenic and non-specific symptoms in early disease stages and limited disease awareness. Similar to observations made in other studies, we have also found a prolonged diagnostic delay [3, 12, 17]. This delay exhibited no significant improvement over time and demonstrated no notable disparity between patients presenting with and without three or more typical red flags. A study conducted in the Netherlands, however, reported diagnostic delay shortening by six months following the implementation of a clinical pathway for CA at their medical centre. They ascribed this improvement to an increased awareness resulting from the dissemination of disease-specific information and regional educational initiatives [3]. Similar results were find in a single center amyloidosis registry of France [11]. However, it remains unclear whether this trend can be extrapolated to a global reduction in diagnostic delays or if it is specific to awareness initiatives within a country and organisation. For instance, a global analysis from the Transthyretin Amyloidosis Outcomes Survey found no improvement of the diagnostic delay between 2007 and 2019 [17]. Furthermore, diverse outcomes are evident in the findings of studies describing the change in symptom to diagnosis duration over time across diverse countries (Supplemental Table S3). Moreover, our research indicates potential variability within healthcare facilities within a single country, as our results diverge from those reported by Brons et al. [3]. These disparities may be attributed to referral bias, as certain studies exclusively incorporated patients referred to a tertiary referral center and therefore potentially miss information of patients who were not referred or referred to a secondary care center. Also, there is evidence that uptake of new methods for the diagnosis of CA is slow beyond specialized centers, resulting in diagnostic delays [18]. These results strike the need for close collaboration between different hospitals, specialists and primary care.

The diagnosis of ATTRwt requires a high index of clinical suspicion which can be raised by identifying well-known diagnostic red flags which is however depending on awareness of the disease. Numerous cardiac and extra-cardiac manifestations serve as distinctive "diagnostic red flags" that warrant thorough investigation during the diagnostic work-up process. Approximately three quarters of ATTR patients presented with echocardiographic LVH (defined as left ventricular wall thickness ≥ 12 mm) preceding ATTR-CM diagnosis at a median of 5.8 [3.3–7.0] years. On the one hand, these findings suggest previous misdiagnosis in a significant proportion of patients and strikes the need to consistently pursue the accurate explanation of the underlying causes of LVH. On the other hand, it is unknown whether this LVH observed years prior to ATTR-CM diagnosis was due to amyloid depositions or represents hypertensive LVH. Interestingly, we observed a high prevalence of systolic arterial hypertension among ATTR patients (54%), which is reported in other studies as well [16, 27]. The study of Gonzalez-Lopez et al. showed that 34% of the CA patients were previously misdiagnosed, of which 35% with hypertensive cardiomyopathy [27]. From a clinical standpoint, a medical history of hypertension in combination with LVH should not rule out CA, particularly when there is a notable discrepancy between the observed wall thickness and the severity of hypertension, or when the patient experiences difficulties tolerating antihypertensive medications. Moreover, the absence of LVH also does not necessarily rule out the presence of CA. Consequently, to achieve an early diagnosis of ATTR-CM, it may be necessary to broaden the scope beyond patients with significant LVH and consider other patient groups as well. The recent T-Amylo study presented a prediction model exhibiting a favourable diagnostic performance for detecting patients with ATTR-CM. This prediction model was additionally validated in various at-risk populations, including HFpEF, hypertensive cardiomyopathy, and aortic valve stenosis. However, it should be noted that this prediction model was exclusively tested in patients with left ventricular wall thickness ≥ 12 mm, potentially limiting its ability to diagnose patients at an early disease stage [28]. Moreover, it is known that measurement of left ventricular wall thickness, exhibits a substantial degree of inter-reader variability, even under ideal conditions [29]. Consequently, relying on LVH for patient diagnosis may still result in a substantial amount of misdiagnoses and/or delayed diagnoses.

Tenosynovial complications, attributed to the progressive infiltration of amyloid into tendon structures, are highly prevalent and precede ATTR-CM diagnosis for many years. These extra-cardiac manifestations represent highly relevant extra-cardiac diagnostic clues and may help identifying patients with ATTR-CM at earlier stages. For instance, in our cohort carpal tunnel syndrome was present in half of ATTR-CM patients preceding diagnosis with a median of 7 years, which is in line with other studies [16, 30]. The study of Cappelli et al. showed that the prevalence of bilateral carpal tunnel syndrome is a lot higher in ATTR patients compared to both hypertrophic cardiomyopathy (2%) and control patients without heart failure (2%) [30]. However, it is important to note that carpal tunnel syndrome is also widely prevalent in the general population. In fact, when utilising an appropriate screening algorithm, only 23% of a preselected group of patients who underwent carpal tunnel release were found to have an amyloid-positive tenosynovial biopsy. Out of the subset of patients with amyloid-positive tenosynovial biopsy, only one (7.1%) individual exhibited cardiac involvement, suggesting that the disease was diagnosed at an early stage. However, it remains uncertain whether these patients will also develop cardiac amyloidosis in the future [31]. The effectiveness of active screening strategies for the early diagnosis of ATTR-CM in patients presenting with tenosynovial manifestations and subsequent follow-up is a topic of ongoing debate. However, it is possible that screening strategies may yield more favourable results when additional red flags or multiple tenosynovial complications are present concurrently. These screening strategies could potentially be augmented with the incorporation of artificial intelligence (AI), given the substantial promise that AI holds in facilitating the identification of early indicators of ATTR-CM. Such integration of AI has the potential to assist in the early-stage diagnosis of ATTR-CM. The effectiveness of active screening strategies, aimed at achieving early diagnosis of ATTR-CM in patients presenting with red flags is yet to be studied. Still, timely diagnosis, referral, and initiation of therapy hold the potential for improving outcomes and quality of life in patients with ATTR-CM [19].

## Study limitations

This study provides real world data on the diagnostic trajectory of CA in the Southern region of the Netherlands. We recognise certain limitations that warrant consideration when interpreting these findings. Firstly, the retrospective design of the study introduces inherent biases, including referral and selection bias and potential missing data. However, the close collaboration of our hospital with the local non-academic hospital(s) warrants minimization of this type of bias. Moreover, reliance on medical records may result in incomplete or inaccurate information, potentially affecting the overall validity of the study. Lastly, the relatively small sample size in this study may restrict the generalizability of the results to larger populations.

## Conclusion

This study highlights the diagnostic trends and clinical characteristics of patients with ATTR-CM. We observed a substantial increase in diagnoses predominantly of the wild type form and a trend towards the use of less invasive diagnostic modalities over time. But, the overall symptom-to-diagnosis duration has remained similar driven by time to referral, despite the presence of typical red flags. Efforts should be focused on reducing pre-diagnostic delays and improving the overall prognosis of amyloidosis patients through timely diagnosis, referral, and initiation of appropriate therapies. Further research is warranted to explore strategies for enhancing disease awareness, improving recognizing at-risk populations and implementing efficient diagnostic pathways to diagnose ATTR-CM patients at an early disease stage.

## Supplementary Information

Below is the link to the electronic supplementary material.

Supplementary file1 (DOCX 19 KB)

Supplementary file2 (DOCX 13 KB)

Supplementary file3 (DOCX 16 KB)

## Data Availability

The data that support the findings of this study are available on request from the corresponding author, AA. The data are not publicly available due to privacy restrictions.

## Abbreviations

AL-CA: Cardiac light chain amyloidosis
ATTR-CM: Transthyretin amyloid cardiomyopathy
ATTRv: Variant transthyretin amyloidosis
ATTRwt: Wild-type transthyretin amyloidosis
CA: Cardiac amyloidosis
LVH: Left ventricular hypertrophy
TTR: Transthyretin

## References

[1] Garcia-Pavia P, Rapezzi C, Adler Y, Arad M, Basso C, Brucato A, Burazor I, Caforio ALP, Damy T, Eriksson U, Fontana M, Gillmore JD, Gonzalez-Lopez E, Grogan M, Heymans S, Imazio M, Kindermann I, Kristen AV, Maurer MS, Merlini G, Pantazis A, Pankuweit S, Rigopoulos AG, Linhart A (2021) Diagnosis and treatment of cardiac amyloidosis. A position statement of the European Society of Cardiology Working Group on Myocardial and Pericardial Diseases. Eur J Heart Fail 23(4):512–526 33826207 10.1002/ejhf.2140

[2] AbouEzzeddine OF, Davies DR, Scott CG, Fayyaz AU, Askew JW, McKie PM, Noseworthy PA, Johnson GB, Dunlay SM, Borlaug BA, Chareonthaitawee P, Roger VL, Dispenzieri A, Grogan M, Redfield MM (2021) Prevalence of transthyretin amyloid cardiomyopathy in heart failure with preserved ejection fraction. JAMA Cardiol 6(11):1267–1274 34431962 10.1001/jamacardio.2021.3070 PMC8387947

[3] Brons M, Muller SA, Rutten FH, van der Meer MG, Vrancken AFJE, Minnema MC, Baas AF, Asselbergs FW, Oerlemans MIFJ (2022) Evaluation of the cardiac amyloidosis clinical pathway implementation: a real-world experience. Eur Heart J Open 2(2):oeac011 35919127 10.1093/ehjopen/oeac011 PMC9242028

[4] Hahn VS, Yanek LR, Vaishnav J, Ying W, Vaidya D, Lee YZJ, Riley SJ, Subramanya V, Brown EE, Hopkins CD, Ononogbu S, Perzel Mandell K, Halushka MK, Steenbergen C Jr, Rosenberg AZ, Tedford RJ, Judge DP, Shah SJ, Russell SD, Kass DA, Sharma K (2020) Endomyocardial biopsy characterization of heart failure with preserved ejection fraction and prevalence of cardiac amyloidosis. JACC Heart Fail 8(9):712–724 32653448 10.1016/j.jchf.2020.04.007 PMC7604801

[5] Lindmark K, Pilebro B, Sundström T, Lindqvist P (2021) Prevalence of wild type transtyrethin cardiac amyloidosis in a heart failure clinic. ESC Heart Fail 8(1):745–749 33205581 10.1002/ehf2.13110 PMC7835553

[6] Mohammed SF, Mirzoyev SA, Edwards WD, Dogan A, Grogan DR, Dunlay SM, Roger VL, Gertz MA, Dispenzieri A, Zeldenrust SR, Redfield MM (2014) Left ventricular amyloid deposition in patients with heart failure and preserved ejection fraction. JACC Heart Fail 2(2):113–122 24720917 10.1016/j.jchf.2013.11.004 PMC3984539

[7] Tanskanen M, Peuralinna T, Polvikoski T, Notkola IL, Sulkava R, Hardy J, Singleton A, Kiuru-Enari S, Paetau A, Tienari PJ, Myllykangas L (2008) Senile systemic amyloidosis affects 25% of the very aged and associates with genetic variation in alpha2-macroglobulin and tau: a population-based autopsy study. Ann Med 40(3):232–239 18382889 10.1080/07853890701842988

[8] Fumagalli C, Zampieri M, Perfetto F, Zocchi C, Maurizi N, Tassetti L, Ungar A, Gabriele M, Nardi G, Del Monaco G, Baldini K, Tomberli A, Tomberli B, Marchionni N, Di Mario C, Olivotto I, Cappelli F (2021) Early diagnosis and outcome in patients with wild-type transthyretin cardiac amyloidosis. Mayo Clin Proc 96(8):2185–2191 34353472 10.1016/j.mayocp.2021.04.021

[9] Maurer MS, Kale P, Fontana M, Berk JL, Grogan M, Gustafsson F, Hung RR, Gottlieb RL, Damy T, González-Duarte A, Sarswat N, Sekijima Y, Tahara N, Taylor MS, Kubanek M, Donal E, Palecek T, Tsujita K, Tang WHW, Yu W-C, Obici L, Simões M, Fernandes F, Poulsen SH, Diemberger I, Perfetto F, Solomon SD, Di Carli M, Badri P, White MT, Chen J, Yureneva E, Sweetser MT, Jay PY, Garg PP, Vest J, Gillmore JD (2023) Patisiran treatment in patients with transthyretin cardiac amyloidosis. N Engl J Med 389(17):1553–1565 37888916 10.1056/NEJMoa2300757 PMC10757426

[10] Maurer MS, Schwartz JH, Gundapaneni B, Elliott PM, Merlini G, Waddington-Cruz M, Kristen AV, Grogan M, Witteles R, Damy T, Drachman BM, Shah SJ, Hanna M, Judge DP, Barsdorf AI, Huber P, Patterson TA, Riley S, Schumacher J, Stewart M, Sultan MB, Rapezzi C (2018) Tafamidis treatment for patients with transthyretin amyloid cardiomyopathy. N Engl J Med 379(11):1007–1016 30145929 10.1056/NEJMoa1805689

[11] Damy T, Zaroui A, de Tournemire M, Kharoubi M, Gounot R, Galat A, Guendouz S, Funalot B, Itti E, Roulin L, Audard V, Fanen P, Leroy V, Poulot E, Belhadj K, Mallet S, Deep Singh Chadah G, Planté-Bordeneuve V, Gendre T, Chevalier X, Guignard S, Bequignon E, Bartier S, Folliguet T, Lemonier F, Audureau E, Tixier D, Canoui-Poitrine F, Lefaucheur JP, Souvannanorath S, Authier FJ, Maupou S, Hittinger L, Molinier-Frenkel V, David JP, Broussier A, Oghina S, Teiger E (2023) Changes in amyloidosis phenotype over 11 years in a cardiac amyloidosis referral centre cohort in France. Arch Cardiovasc Dis 116(10):433–446 37640624 10.1016/j.acvd.2023.07.003

[12] Dang D, Fournier P, Cariou E, Huart A, Ribes D, Cintas P, Roussel M, Colombat M, Lavie-Badie Y, Carrié D, Galinier M, Lairez O (2020) Gateway and journey of patients with cardiac amyloidosis. ESC Heart Fail 7(5):2418–2430 32588554 10.1002/ehf2.12793 PMC7524246

[13] López-Sainz Á, Hernandez-Hernandez A, Gonzalez-Lopez E, Domínguez F, Restrepo-Cordoba MA, Cobo-Marcos M, Gómez-Bueno M, Hernandez-Perez FJ, Oteo JF, Mirelis JG, Cavero MA, Moñivas V, Mingo Santos S, de Haro-Del Moral FJ, Krsnik I, Salas C, Bornstein B, Briceño A, López JA, Vázquez J, Alonso-Pulpón L, Segovia J, Garcia-Pavia P (2021) Clinical profile and outcome of cardiac amyloidosis in a Spanish referral center. Rev Esp Cardiol (Engl Ed) 74(2):149–158 32317158 10.1016/j.recesp.2019.12.017

[14] Nativi-Nicolau JN, Karam C, Khella S, Maurer MS (2022) Screening for ATTR amyloidosis in the clinic: overlapping disorders, misdiagnosis, and multiorgan awareness. Heart Fail Rev 27(3):785–793 33609196 10.1007/s10741-021-10080-2 PMC9033715

[15] Oerlemans M, Rutten KHG, Minnema MC, Raymakers RAP, Asselbergs FW, de Jonge N (2019) Cardiac amyloidosis: the need for early diagnosis. Neth Heart J 27(11):525–536 31359320 10.1007/s12471-019-1299-1 PMC6823341

[16] Debonnaire P, Claeys M, De Smet M, Trenson S, Lycke M, Demeester C, Van Droogenbroeck J, De Vriese AS, Verhoeven K, Vantomme N, Van Meirhaeghe J, Willandt B, Lambert M, de Paepe P, Delanote J, De Geeter F, Tavernier R (2022) Trends in diagnosis, referral, red flag onset, patient profiles and natural outcome of de novo cardiac amyloidosis and their multidisciplinary implications. Acta Cardiol 77(9):791–804 34565298 10.1080/00015385.2021.1976450

[17] Nativi-Nicolau J, Siu A, Dispenzieri A, Maurer MS, Rapezzi C, Kristen AV, Garcia-Pavia P, LoRusso S, Waddington-Cruz M, Lairez O, Witteles R, Chapman D, Amass L, Grogan M (2021) Temporal trends of wild-type transthyretin amyloid cardiomyopathy in the transthyretin amyloidosis outcomes survey. JACC CardioOncol 3(4):537–546 34729526 10.1016/j.jaccao.2021.08.009 PMC8543133

[18] Rozenbaum MH, Large S, Bhambri R, Stewart M, Whelan J, van Doornewaard A, Dasgupta N, Masri A, Nativi-Nicolau J (2021) Impact of delayed diagnosis and misdiagnosis for patients with transthyretin amyloid cardiomyopathy (ATTR-CM): a targeted literature review. Cardiol Ther 10(1):141–159 33877591 10.1007/s40119-021-00219-5 PMC8126532

[19] Kittleson MM, Ruberg FL, Ambardekar AV, Brannagan TH, Cheng RK, Clarke JO, Dember LM, Frantz JG, Hershberger RE, Maurer MS, Nativi-Nicolau J, Sanchorawala V, Sheikh FH (2023) 2023 ACC expert consensus decision pathway on comprehensive multidisciplinary care for the patient with cardiac amyloidosis. J Am Coll Cardiol 81(11):1076–1126 36697326 10.1016/j.jacc.2022.11.022

[20] Castano A, Haq M, Narotsky DL, Goldsmith J, Weinberg RL, Morgenstern R, Pozniakoff T, Ruberg FL, Miller EJ, Berk JL, Dispenzieri A, Grogan M, Johnson G, Bokhari S, Maurer MS (2016) Multicenter study of planar technetium 99m pyrophosphate cardiac imaging: predicting survival for patients with ATTR cardiac amyloidosis. JAMA Cardiol 1(8):880–889 27557400 10.1001/jamacardio.2016.2839

[21] Henkens M, Weerts J, Verdonschot JAJ, Raafs AG, Stroeks S, Sikking MA, Amin H, Mourmans SGJ, Geraeds CBG, Sanders-van Wijk S, Barandiarán Aizpurua A, Uszko-Lencer N, Krapels IPC, Wolffs PFG, Brunner HG, van Leeuwen REW, Verhesen W, Schalla SM, van Stipdonk AWM, Knackstedt C, Li X, Abdul Hamid MA, van Paassen P, Hazebroek MR, Vernooy K, Brunner-La Rocca HP, van Empel VPM, Heymans SRB (2022) Improving diagnosis and risk stratification across the ejection fraction spectrum: the Maastricht Cardiomyopathy registry. ESC Heart Fail 9(2):1463–1470 35118823 10.1002/ehf2.13833 PMC8934928

[22] Lang RM, Badano LP, Mor-Avi V, Afilalo J, Armstrong A, Ernande L, Flachskampf FA, Foster E, Goldstein SA, Kuznetsova T, Lancellotti P, Muraru D, Picard MH, Rietzschel ER, Rudski L, Spencer KT, Tsang W, Voigt JU (2015) Recommendations for cardiac chamber quantification by echocardiography in adults: an update from the American Society of Echocardiography and the European Association of Cardiovascular Imaging. J Am Soc Echocardiogr 28(1):1-39.e14 25559473 10.1016/j.echo.2014.10.003

[23] Gillmore JD, Damy T, Fontana M, Hutchinson M, Lachmann HJ, Martinez-Naharro A, Quarta CC, Rezk T, Whelan CJ, Gonzalez-Lopez E, Lane T, Gilbertson JA, Rowczenio D, Petrie A, Hawkins PN (2018) A new staging system for cardiac transthyretin amyloidosis. Eur Heart J 39(30):2799–2806 29048471 10.1093/eurheartj/ehx589

[24] Maurer MS, Elliott P, Comenzo R, Semigran M, Rapezzi C (2017) Addressing common questions encountered in the diagnosis and management of cardiac amyloidosis. Circulation 135(14):1357–1377 28373528 10.1161/CIRCULATIONAHA.116.024438 PMC5392416

[25] Tini G, Milani P, Zampieri M, Caponetti AG, Fabris F, Foli A, Argirò A, Mazzoni C, Gagliardi C, Longhi S, Saturi G, Vergaro G, Aimo A, Russo D, Varrà GG, Serenelli M, Fabbri G, De Michieli L, Palmiero G, Ciliberti G, Carigi S, Sessarego E, Mandoli GE, Ricci Lucchi G, Rella V, Monti E, Gardini E, Bartolotti M, Crotti L, Merli E, Mussinelli R, Vianello PF, Cameli M, Marzo F, Guerra F, Limongelli G, Cipriani A, Perlini S, Obici L, Perfetto F, Autore C, Porto I, Rapezzi C, Sinagra G, Merlo M, Musumeci B, Emdin M, Biagini E, Cappelli F, Palladini G, Canepa M (2023) Diagnostic pathways to wild-type transthyretin amyloid cardiomyopathy: a multicentre network study. Eur J Heart Fail 25(6):845–853 36907828 10.1002/ejhf.2823

[26] World Health Organization DoDaADoD (2020) WHO methods and data sources for country-level causes of death 2000–2019. Global Health Estimates Technical Paper WHO/DDI/DNA/GHE/20202

[27] González-López E, Gagliardi C, Dominguez F, Quarta CC, de Haro-Del Moral FJ, Milandri A, Salas C, Cinelli M, Cobo-Marcos M, Lorenzini M, Lara-Pezzi E, Foffi S, Alonso-Pulpon L, Rapezzi C, Garcia-Pavia P (2017) Clinical characteristics of wild-type transthyretin cardiac amyloidosis: disproving myths. Eur Heart J 38(24):1895–1904 28329248 10.1093/eurheartj/ehx043

[28] Arana-Achaga X, Goena-Vives C, Villanueva-Benito I, Solla-Ruiz I, Rengel Jimenez A, Gaspar TI, Urreta-Barallobre I, Barge-Caballero G, Seijas-Marcos S, Cabrera E, Garcia-Pavía P, Basurte Elorz MT, Ayestarán NM, Sierra LT, Robledo Iñarritu M, Lozano-Bahamonde A, Escolar-Perez V, Gómez-Ramírez C, Alzola E, Andrés RN, Francisco Matias JL, Limeres Freire J, Armengou Arxe A, Negre Busó M, Piqueras-Flores J, Martínez-Del Río J, Onaindia Gandarias JJ, Rodriguez Sanchez I, Querejeta Iraola R (2023) Development and validation of a prediction model and score for transthyretin cardiac amyloidosis diagnosis: T-Amylo. JACC Cardiovasc Imaging 16(12):1567–1580 37389511 10.1016/j.jcmg.2023.05.002

[29] Captur G, Manisty CH, Raman B, Marchi A, Wong TC, Ariga R, Bhuva A, Ormondroyd E, Lobascio I, Camaioni C, Loizos S, Bonsu-Ofori J, Turer A, Zaha VG, Augutsto JB, Davies RH, Taylor AJ, Nasis A, Al-Mallah MH, Valentin S, Arenaza DPd, Patel V, Westwood M, Petersen SE, Li C, Tang L, Nakamori S, Nezafat R, Kwong RY, Ho CY, Fraser AG, Watkins H, Elliott PM, Neubauer S, Lloyd G, Olivotto I, Nihoyannopoulos P, Moon JC (2021) Maximal wall thickness measurement in hypertrophic cardiomyopathy. JACC Cardiovasc Imaging 14(11):2123–2134 34147459 10.1016/j.jcmg.2021.03.032

[30] Cappelli F, Zampieri M, Fumagalli C, Nardi G, Del Monaco G, Matucci Cerinic M, Allinovi M, Taborchi G, Martone R, Gabriele M, Ungar A, Moggi Pignone A, Marchionni N, Di Mario C, Olivotto I, Perfetto F (2021) Tenosynovial complications identify TTR cardiac amyloidosis among patients with hypertrophic cardiomyopathy phenotype. J Intern Med 289(6):831–839 33615623 10.1111/joim.13200

[31] Gannon NP, Ward CM (2023) Results of Implementation of Amyloidosis Screening for Patients Undergoing Carpal Tunnel Release. J Hand Surg Am 47:517–525 10.1016/j.jhsa.2022.09.005 36646584

