# Supplementary material for: Changes in the diagnostic trajectory of transthyretin cardiac amyloidosis over six years
Source: Heart Vessels. 2024 May 6;39(10):857–66. doi: 10.1007/s00380-024-02408-3 (PMC11405426; doi:10.1007/s00380-024-02408-3)
Supplement: Supplementary file 1 — Supplementary file1 (DOCX 19 KB) [file 380_2024_2408_MOESM1_ESM.docx]

| **Characteristic** | **Valid, *n*** | **ATTRwt n = 59 (91%)** | **Valid, *n*** | **ATTRv n = 6 (9%)** | **p-value** |
| --- | --- | --- | --- | --- | --- |
| Male sex | 59 | 54 (92%) | 6 | 2 (33%) | 0.002* |
| Age (years) | 59 | 78 [75-82] | 6 | 71 [65-74] | 0.015* |
| **Medical history:** |  |  |  |  |  |
| Diabetes mellitus type II | 59 | 10 (17%) | 6 | 1 (17%) | 1.000 |
| Kidney disease | 56 | 25 (42%) | 6 | 2 (33%) | 0.693 |
| Atrial fibrillation/flutter | 59 | 42 (71%) | 6 | 2 (33%) | 0.080 |
| Hypertension | 59 | 42 (71%) | 6 | 2 (33%) | 0.403 |
| Significant CAD | 56 | 19 (32%) | 6 | 1 (17%) | 0.655 |
| Stroke | 59 | 6 (10%) | 6 | 0 (0%) | 1.000 |
| **NYHA class** | 55 |  | 6 |  | 0.602 |
| NYHA I |  | 1 (2%) |  | 1 (17%) |  |
| NYHA II |  | 30 (51%) |  | 3 (50%) |  |
| NYHA III |  | 23 (39%) |  | 1 (17%) |  |
| NYHA IV |  | 1 (2%) |  | 1 (17%) |  |
| **Perugini grade:** | 50 |  | 3 |  | 0.433 |
| Perugini 0 |  | 1 (2%) |  | 0 (0%) |  |
| Perugini 1 |  | 1 (2%) |  | 1 (33%) |  |
| Perugini 2 |  | 27 (46%) |  | 1 (33%) |  |
| Perugini 3 |  | 21 (36%) |  | 1 (33%) |  |
| **Electrocardiography:** |  |  |  |  |  |
| Heart rate (beats/min) | 55 | 72 [64-84] | 6 | 70 [59-89] | 0.827 |
| Abnormal axis | 50 | 28 (48%) | 4 | 2 (33%) | 1.000 |
| Micro voltage | 45 | 25 (42%) | 4 | 1 (17%) | 0.330 |
| High voltage | 45 | 0 (0%) | 4 | 0 (0%) | 1.000 |
| QRS duration (ms) | 50 | 108 [92-142] | 4 | 98 [88-111] | 0.322 |
| QRS ≥ 120ms | 50 | 19 (32%) | 4 | 1 (17%) | 0.656 |
| PQ duration (ms) | 26 | 209 [195-247] | 3 | 166 [159-173] | 0.018* |
| PQ ≥ 200ms | 26 | 17 (29%) | 3 | 0 (0%) | 0.170 |
| Pseudo infarct pattern | 42 | 20 (34%) | 4 | 4 (67%) | 0.110 |
| Delayed R wave propagation | 37 | 23 (39%) | 4 | 4 (67%) | 0.280 |
| Abnormal repolarization | 49 | 41 (70%) | 4 | 3 (50%) | 0.536 |
| **Echocardiography:** |  |  |  |  |  |
| LVEF (%) | 59 | 50 [41-56] | 6 | 52 [42-58] | 0.594 |
| IVSd (mm) | 57 | 14 [12-16] | 6 | 13 [10-17] | 0.621 |
| PWd (mm) | 56 | 13 [11-15] | 6 | 11 [9-14] | 0.200 |
| LVEDd (mm) | 45 | 45 [41-51] | 6 | 44 [42-46] | 0.491 |
| LVMI (g/m²) | 37 | 122 [101-146] | 6 | 113 [72-151] | 0.881 |
| LAVI (ml/m^2^) | 45 | 55 [47-61] | 6 | 29 [26-56] | 0.599 |
| e’ septal (cm/s) | 40 | 5.0 [3.9, 5.9] | 4 | 4.3 [3.8, 5.4] | 0.744 |
| e’ lateral (cm/s) | 40 | 6.4 [5.7, 7.8] | 4 | 7.2 [6.6, 8.1] | 0.567 |
| E/e’ average | 37 | 14.4 [10.5, 17.9] | 4 | 9.9 [8.2, 13.2] | 0.262 |
| TR velocity (m/s) | 41 | 2.4 [2.2, 2.8] | 5 | 2.5 [2.2, 2.5] | 0.818 |
| **Laboratory testing:** |  |  |  |  |  |
| eGFR (ml/min/1.73m^2^) | 53 | 58.7 [40.8- 74.8] | 6 | 65.4 [57.2- 68.7] | 0.401 |
| hsTnT (ng/l) | 39 | 49 [37- 68] | 5 | 26 [9- 128] | 0.618 |
| NT-proBNP (pg/ml) | 52 | 2419 [1328-3983] | 5 | 4102 [68-4110] | 1.000 |
