# Supplementary material for: Changes in the diagnostic trajectory of transthyretin cardiac amyloidosis over six years
Source: Heart Vessels. 2024 May 6;39(10):857–66. doi: 10.1007/s00380-024-02408-3 (PMC11405426; doi:10.1007/s00380-024-02408-3)
Supplement: Supplementary file 2 — Supplementary file2 (DOCX 13 KB) [file 380_2024_2408_MOESM2_ESM.docx]

| **Gillmore prognostic stage** | **ATTRwt n = 59** | **ATTRv n = 6** | **p-value**  0.976 |
| --- | --- | --- | --- |
| STAGE I | 21 (36%) | 2 (33%) |  |
| STAGE IIa | 8 (14%) | 0 (0%) |  |
| STAGE IIb | 14 (24%) | 3 (50%) |  |
| STAGE III | 8 (14%) | 0 (0%) |  |
