# Supplementary material for: Changes in the diagnostic trajectory of transthyretin cardiac amyloidosis over six years
Source: Heart Vessels. 2024 May 6;39(10):857–66. doi: 10.1007/s00380-024-02408-3 (PMC11405426; doi:10.1007/s00380-024-02408-3)
Supplement: Supplementary file 3 — Supplementary file3 (DOCX 16 KB) [file 380_2024_2408_MOESM3_ESM.docx]

| Author | Inclusion period | Stuyd desing | Symptoms | Included patients | Results |
| --- | --- | --- | --- | --- | --- |
| Nativi-Nicolau et al. (2021) | 2007-2020 | Multicenter, global observational cohort study (15 countries) | Cardiac and non cardicac | 1.069 ATTRwt patients  95% male;  Mean age 77.0 ± 7.2 years | No significantly reduction in symptom-diagnosis duration. A median symptom-diagnosis duration of:  63 months in 2015,  68 months in 2016,  62 months in 2017,  73 months in 2018,  72 months in 2019 |
| Damy et al. (2023) | 2010-2020 | Single referral center (France), retrospective, observational cohort study | Cardiac | 684 ATTRwt patients  85% male  Median age 81.8 years [77.1-87.2] | Significant reduction in symptom-diagnosis duration (p=0.020). A median symptom-diagnosis duration of:  5 months in 2010-2012  27 months in 2013-2014  26 months in 2015-2016  17 months in 2017-2018  12 month in 2019-2020 |
| Brons et al. (2022) | 2007-2020 | Single referral center (Netherlands), retrospective, observational cohort study | Cardiac | 43 ATTR patients  86% male  Mean age 73.0 ± 6.0 years | Significant reduction in symptom-diagnosis duration (p<0.001). A median symptom-diagnosis duration of:  18 months in 2007-2018  8 months in 2019-2020 |
| Debonnaire et al. (2023) | 2012-2021 | Single referral center (Belgium), retrospective, observational cohort study | Cardiac | 114 ATTR patients  76% male  Median age 81 years [58–95] | Overall (AL+ATTR) no significant reduction in symptom-diagnosis duration (p=0.664)  9 months in 2012-2016  8 months in 2017-2021 |
